# MiR-34a-5p and miR-452-5p: The Novel Regulators of Pancreatic Endocrine Dysfunction in Diabetic Zucker Rats?

**DOI:** 10.7150/ijms.62843

**Published:** 2021-07-11

**Authors:** Tong Su, Jiejun Hou, Tonghua Liu, Pei Dai, LingLing Qin, Lei Ding, Yan Hu, Xiangyu Guo

**Affiliations:** 1 Dongfang Hospital of Beijing University of Chinese Medicine, Beijing, Beijing 100078, China.; 2 Affiliated hospital of Shan'xi University of Chinese Medicine, Xianyang, Shanxi 712000, China.; 3 Beijing University of Chinese Medicine, Beijing, Beijing 100029, China.

**Keywords:** Type 2 diabetes, Pancreatic endocrine dysfunction, miR-34a-5p, miR-452-5p, Biomarkers

## Abstract

**Objective:** The pancreatic endocrinal system dominates the regulation of blood glucose levels in vivo, and the dysfunction of pancreatic endocrine β-cells is a major cause of the occurrence and development of Type 2 diabetes (T2D). Although microRNA (miRNA) have been found to be key regulators of pancreatic β-cells proliferation, differentiation and apoptosis, the underlying mechanism remains enigmatic. The aim of this study was to identify several novel miRNAs which might be involved in the etiopathogenesis of diabetic β-cells dysfunction.

**Methods:** The miRNA expression profiles in the pancreas of high-fat diet (HFD) fed Zucker diabetic fatty (ZDF) rats and Zucker lean (ZL) rats feed with normal-fat diet (NFD) were detected by using miRNA microarray chip, and individually verified the most significant factors by quantitative real-time polymerase chain reaction (qRT-PCR) assay. The Gene Ontology (GO) and Kyoto Encyclopedia of Genes and Genomes (KEGG) enrichment analyses were used to predict the target genes related to each of the identified miRNAs and the functions of these target genes in different metabolic signaling pathways.

**Results:** Compared with the ZL rats, a total of 24 differentially expressed miRNAs were detected in ZDF rats. Among which miR-34a-5p and miR-452-5p were the most significantly up-regulated and down-regulated respectively. These miRNAs have not been reported in rats' pancreas before. By GO and KEGG enrichment analyses, we found that miR-34a-5p could negatively regulate pancreatic β-cell proliferation through the involvement of Wnt signaling pathway. In addition, it was also found to regulate insulin secretion through the insulin signaling pathway to modulate blood glucose levels. At the same time, miR-452-5p was found to positively regulate the activity of the key rate-limiting enzyme branched-chain α-keto acid dehydrogenase-β (BCKDHB) in the catabolism of branched chain amino acids (BCAA), leading to mitochondrial dysfunction in pancreatic β-cells.

**Conclusions:** miR-34a-5p and miR-452-5p were identified as the novel regulators of pancreatic endocrine dysfunction. These miRNAs might have the potential to be utilized as the new predictive biomarkers for the diagnosis of the occurrence and development of T2D, as well as the therapeutic targets for T2D treatment.

## Introduction

Insulin secreted by pancreatic endocrine β-cells is one of the major hormones that regulate glucose concentrations in the human body. Hence, dysregulation of insulin secretion is closely related to the occurrence and progression of type 2 diabetes (T2D). According to UK Prospective Diabetes Study (UKPDS)^1^, ^2^, Homeostasis Model Assessment (HOMA) index of β-cell function (HOMA-β) indicates that β-cell function is reduced by 50% in T2D patients, and it exhibits progressive decline at a rate of approximately 5% per year. It is frustrating that the continuous loss of β-cell function and mass cannot be stopped and/or reversed by available therapeutic intervention regimens (dieting, exercise, or even hypoglycemic drugs). Therefore, the functional modulation of pancreatic β-cells has always been a central focus in diabetes research. Although apoptosis and dedifferentiation are considered to be the main causes of β-cell dysfunction leading to T2D, however, there are still many yet unexplored mechanisms, including modulation of β-cells' gene expression, RNA processing and maturation, as well as the activation of corresponding signal transduction pathways.

MicroRNA (miRNA) is a class of small non-coding RNA molecules with 21-23 nt in length^3^. Since the discovery of evolutionarily conserved pancreatic β cell-specific miR-375, regulating insulin secretion, in 2004 by Matthew N. Poy et al.^4^, a large number of miRNAs have been identified that are involved in the onset of diabetes. For example, Nadine Baroukh et al.^5^ has reported that miR-124a plays a role in the formation of pancreatic cells. The primary function of these miRNAs includes facilitating the formation of an RNA-induced silencing complex (RISC) to regulate the stability of target mRNAs by binding to their 3'-untranslated region (UTR), thereby further controlling the protein levels of those particular target genes^6^. These functions of those miRNAs can directly affect β-cell proliferation, differentiation, maturation, and apoptosis^7^. Therefore, inhibiting or accelerating the expression of the regulatory miRNAs in pancreatic β-cells can be a new therapeutic approach to treat diabetic patients^8^.

The genotype-phenotype interaction of glucose metabolism in T2D is very complex and greatly varies across the species^9^. Importantly, most current research studies on pancreatic miRNA profiles have been mainly conducted on mice and humans. Furthermore, there has been a lack of miRNAs expression profiling analyses of rats. In this study, we aimed to analyze the miRNA expression profiles in the pancreatic cells of the high-fat-diet (HFD) induced Zucker diabetic fatty (ZDF, fa/fa) rats and the normal-fat-diet (NFD) fed Zucker lean (ZL, +/fa) rats using miRNA microarray chip technology, and single validated selected miRNAs using quantitative real-time polymerase chain reaction (qRT-PCR) to identify novel miRNAs which may be involved in the etiopathogenesis of pancreatic endocrine β-cell dysfunction of T2D.

## Materials and Methods

### Animals and pancreatic tissues collection

7-weeks-old male ZDF rats and ZL rats with weights ranging from180g to 200g, were purchased from Beijing Vital River Laboratory Animal Technologies (Beijing, China). All rats were housed in a specific pathogen-free facility in the Beijing University of Chinese Medicine. After one week of adaptive feeding with regular food, ZDF rats were fed a (HFD) as the experimental group (n=5), and ZL rats were fed a (NFD) as a control group (n=5) for about 4 weeks. Body weight and fasting plasma glucose levels were measured routinely every week. The animal experiment protocol was approved by Beijing University of Chinese Medicine.

### Measurement of plasma insulin, triglycerides, cholesterol, and FFA

Rats were fasted for 12h before being sacrificed, and blood samples were then obtained from individual rats for the measurement of the concentrations of fasting plasma insulin, triglycerides, cholesterol, and free fatty acids (FFA). Specific ELISA kits (Wako Pure Chemical Industries, Osaka, Japan) were used to measure the concentrations of plasma insulin following manufacturer's protocol. The detection values of triglycerides, cholesterol, and FFA were read on an AU400 Biochemical Analyzer. Then the average was calculated from triplicate values for each of the parameters.

### Pancreatic tissues collection and immunofluorescence staining

After the rats were sacrificed, part of the pancreatic tissues was quickly removed and placed in the refrigerator at -80℃ for a future test. The other portion was fixed in 4% paraformaldehyde for 24h, then embedded in paraffin, sliced into 5μm sections, deparaffinized with conventional xylene and gradient alcohol method. After that, the sections were washed with 1×PBS and treated with 0.01M sodium citrate buffer and placed in a microwave oven for antigen retrieval. While they were being cooled, Triton X-100 was used to permeabilize the cell membrane for 15min at room temperature. Then sections were blocked with 10% goat serum for 30min at room temperature. Then the sections were added with the mixed primary antibodies [Insulin Antibody (1:100 dilution, Boster Biological Technology, USA), Glucagon Polyclonal Antibody (1:100 dilution, Bioss Antibodies, China), mixed in 1:1 ratio] and incubated overnight at 4℃. The next day, slides were placed for 1h to restore room temperature, following staining with mixed fluorescent secondary antibodies [Fluorescein-Conjugated AffiniPure Goat Anti-Mouse IgG (H+L) (1:100 dilution, ZSGB-BIO, China), Fluorescein-Conjugated AffiniPure Goat Anti-Rabbit IgG (H+L) (1:100 dilution, ZSGB-BIO, China), mixed in 1:1 ratio] were added, the sections were then incubated at room temperature for 1h in the dark, and the nucleus was subsequently stained with DAPI. At the final step, the fluorescence quenching agent was added, and sections were mounted with a cover slip. A fluorescence microscope was used to observe the results and capture the images.

### MicroRNA isolation and microarray analysis

MiRNeasy Mini Kit (Qiagen, Germany) were used to extract total RNA from the pancreas tissue samples for comparison. First, the 5'end phosphate group of RNA was removed by using the dephosphorylation reaction mixture. After the sample denaturation and labeling reaction, the fluorescent dye was conjugated to the 3'end of RNA under the action of RNA ligase, then the hybridization reaction solution was added. After that, added samples and install on the Agilent`s miRNA chip platform, according to the manufacturer's instructions. The hybridization was carried out at 55°C and 20rpm for 20h. After washing and scanning, the chips were scanned with Agilent Feature Extraction (AFE) software version 10.7.1.1, and the original data were normalized as suggested.

### Prediction and functional analysis of microRNA targets

TargetScan (http://www.targetscan.org/) and miRDB (http://mirdb.org/miRDB/) were used for target gene prediction. For each set of predicted target genes, the function and pathway enrichment analyses were performed based on the Gene Ontology (GO, http://www.geneontology.org) and Kyoto Encyclopedia of Genes and Genomes (KEGG, http://www.kegg.jp/kegg/pathway.html) databases at the same time. All the data with P<0.05 were considered statistically significant. More than 2 times of high expression or 0.5 times of low expression was considered highly significant.

### qRT-PCR

Mircute miRNA isolation Kit (Tiangen, Beijing) was used to extract total RNA from pancreatic tissues. Then the total RNA was reversely transcribed into cDNA with GoScript™ Reverse Transcription System Kit (Promega, USA) according to the manufacturers' instructions. For cDNA preparation, 1μg of the total RNA was incubated at 70℃ for 10min, and then the reaction tubes were kept on the ice after giving a short centrifugation. The thermal cycling profile for the reverse transcription reaction was as follows: 25℃ for 10min, 42℃ for 1h, 95℃ for 5min, and 4℃ for 5min. The relative levels of miR-34a-5p and miR-452-5p transcripts along with the internal control U6 small RNA were determined by qRT-PCR using GoTaq® qPCR Master Mix (Promega, USA) on the Applied Biosystems 7500 Real Time PCR System. The reaction conditions were as follow: 95℃ for 2min to pre-degeneration, then 95℃ for 15s, 60℃ for 1min and 72℃ for 10min loop 40 times. The primer sequences used in this qRT-PCR assay are listed in Table S3.

### Statistical analysis

SPSS 22.0 software was used to analyze the data. All data are represented as mean ± standard deviation (SD). The comparison of body weight, fasting glucose, plasma insulin, triglycerides, cholesterol, and FFA between two groups was performed using the Mann-Whitney U test, and the qRT-PCR data was performed with the unpaired student t-test method. Experimental results with P <0.05 were considered statistically significant.

## Results

### Effects of HFD-feed on ZDF rats

**Figure 1:**
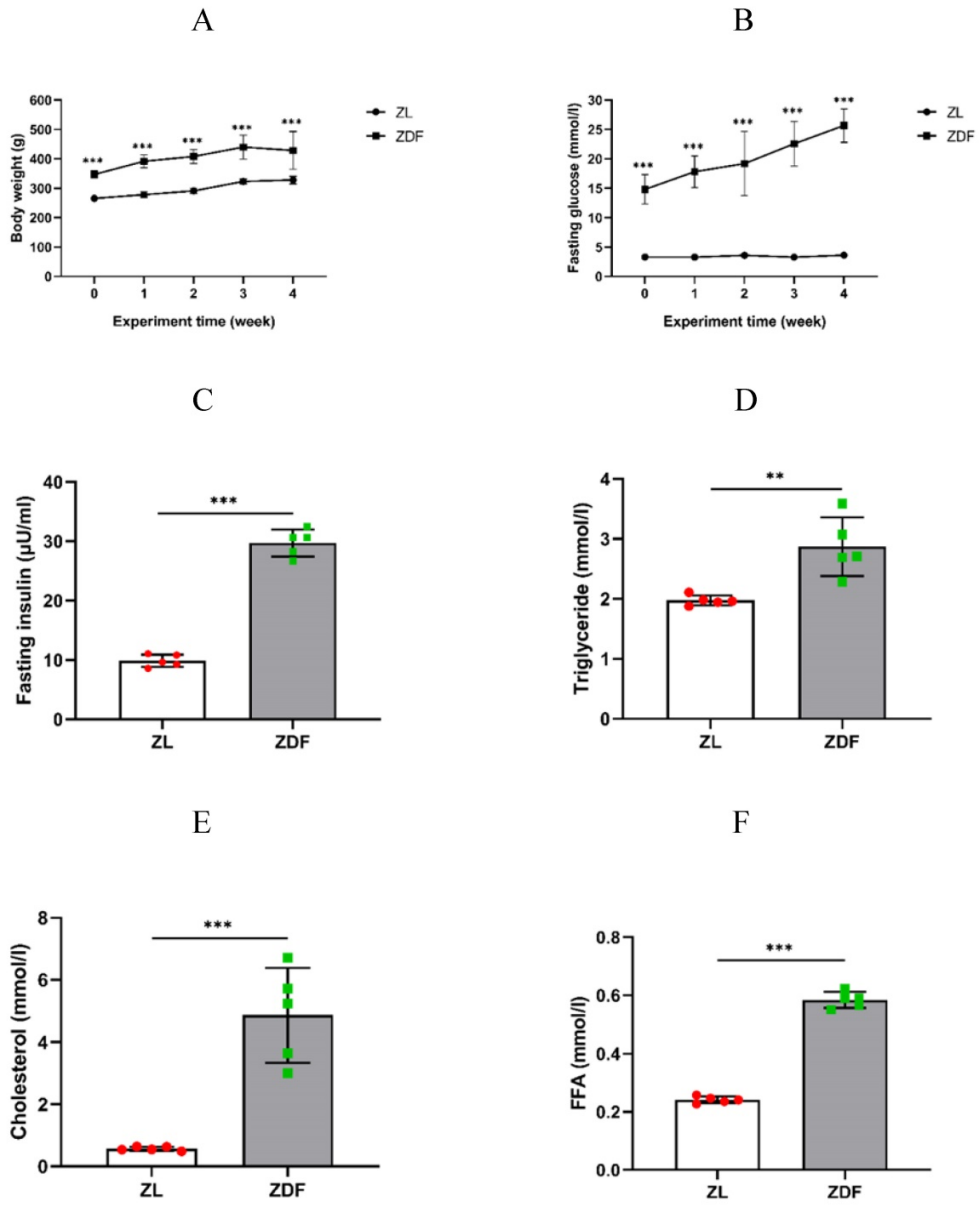
The effect of HFD-feed on ZDF rats. Rats' body weights (A) and fasting blood glucose (B) were measured on weekly basis. The levels of fasting plasma insulin (C), triglyceride (D), cholesterol (E) and FFA (F) were measured after 4 weeks feeding. The data of each group of rats (n=5 per group) are represented as mean±SD. Statistical significances between ZL and ZDF groups are indicated by ^*^P<0.05, ^**^P<0.01, ^***^P<0.001.

Body weight and fasting blood glucose levels of the control and experimental rats were routinely measured every week to confirm that the average body weight of the ZDF rats was obviously higher than that of the control group rats (P<0.001 of any week's data, Figure 1A, Table S1). The fasting blood glucose levels of the ZDF rats significantly increased and within diabetic range compared to that in control rats (P<0.001 of any week's data, Figure 1B, Table S2). Our results showed that the levels of plasma insulin (9.87±1.05 vs. 29.72±2.27 μU/ml, P<0.001, Figure 1C), triglyceride (1.97±0.08 vs. 2.87±0.49 mmol/l, P<0.01, Figure 1D), cholesterol (0.57±0.07 vs. 4.86±1.52 mmol/l, P<0.001, Figure 1E), and FFA content (0.24±0.01 vs. 0.58±0.03 mmol/l, P<0.001, Figure 1F) were significantly higher in ZDF rats compared to those in ZL rats.

### Diabetes-induced pathological changes of pancreas morphology

**Figure 2:**
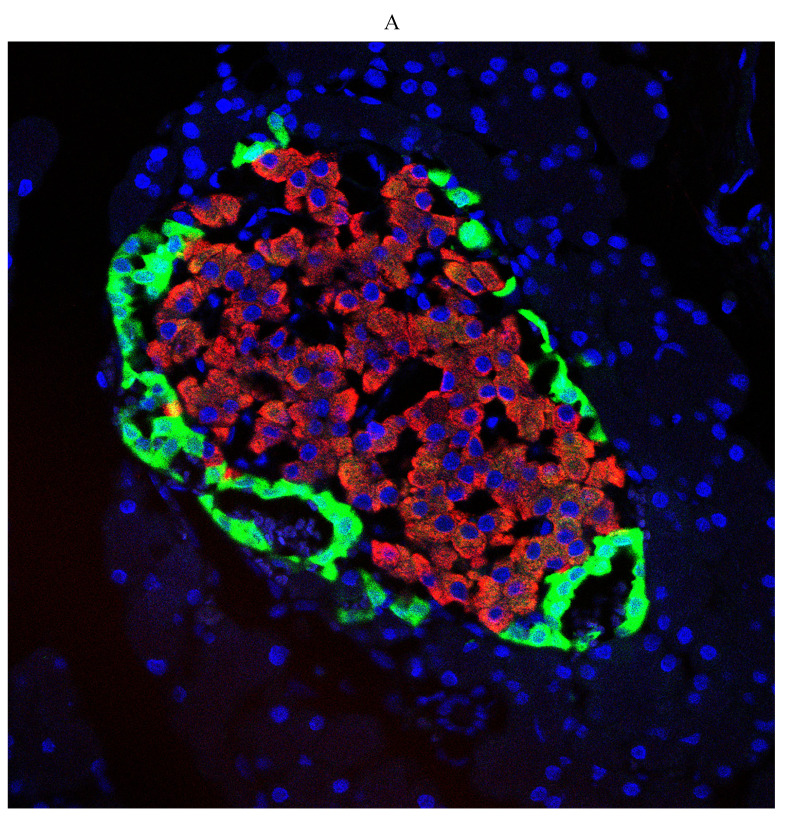

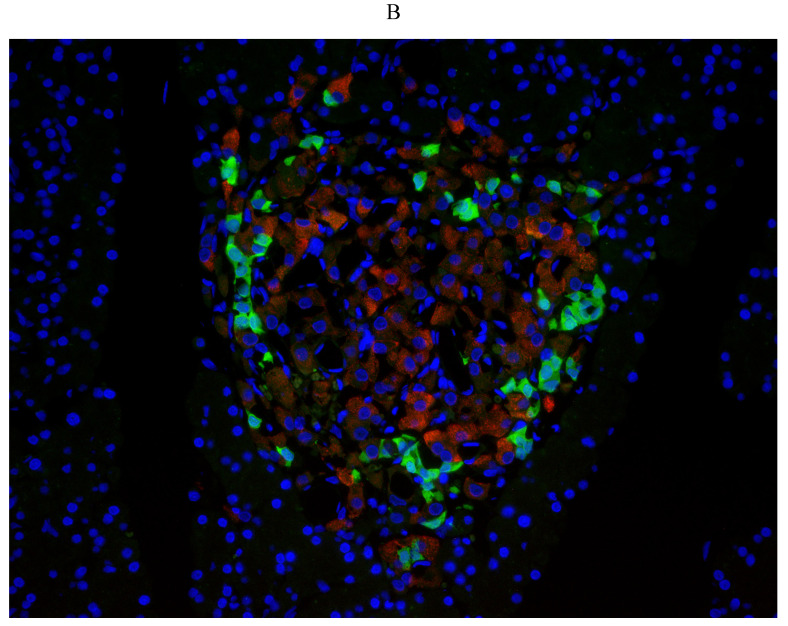
Pathological changes in the pancreas of the diabetic and normal rats. A) Represented the IF image of pancreatic islet cells of the control group. B) Represented IF image exhibiting the pathological changes in pancreatic islet cells in ZDF rats. Here, the red color indicated Insulin, green indicated Glucagon and blue indicated the DAPI staining of the nucleus. Scale bars, 20 μm; n=5 for each group.

First, we examined diabetes-induced morphological differences in pancreas between the control and diabetic rats by immunofluorescence (IF) staining with tissue-specific cell surface markers (Figure 2), which revealed that the pancreatic islets of normal ZL rats had regular morphology and clear boundaries, the α cells secreting glucagon were distributed uniformly around the islets, the population of β cells was normal and distributed in the center of the islets, and the level of insulin secretion was higher (Figure 2A). While the pancreatic islets of diabetic ZDF rats exhibited atrophy, with irregular tissue morphology and unclear borders, not only the number of β cells was significantly reduced, but also the level of secreted insulin was insufficient along with the abnormal distribution of α cells and β cells (Figure 2B).

### Cluster analysis of the differentially microRNAs in pancreas of ZDF rats

**Figure 3:**
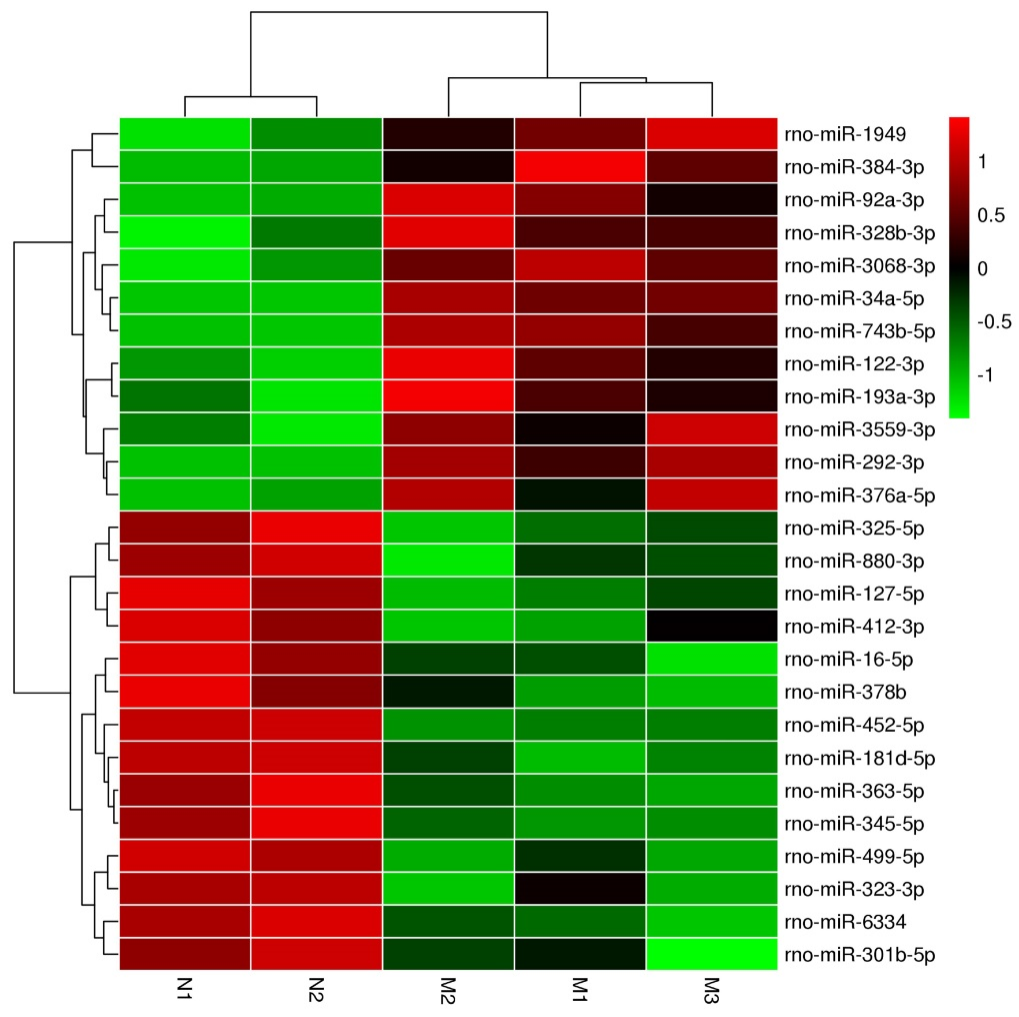
The heatmap analysis of differentially expressed miRNAs. In the cluster heatmap expression analysis, the red color indicated high expression level, green indicated low expression, and black indicated that there was no expression. The darker the color looks, the higher or lower the expression level is. Number N1 and Number N2 are the control (ZL) samples, number M1, Number M2 and NumberM3 are the experimental (ZDF) samples.

Our cluster analysis of miRNA profiles showed that the expression levels of more than 700 miRNAs were altered in the pancreas of ZDF rats compared to that in ZL rats. Amongst the differentially expressed miRNAs, 10 miRNAs were highly expressed, while another 14 miRNAs exhibited significantly reduced expression levels, especially miR-34a-5p and miR-452-5p, which showed the most significant difference according to the heat map (Figure 3 and Table 1). These two miRNAs have also been previously reported in other diseases, such as acute ischemic stroke, diabetic kidney disease, and gestational diabetes, but there has been no report suggesting their roles in diabetic pancreatic islet dysfunction. Hence, it is crucial to identify the functional consequences of differential expression of these miRNAs in diabetes.

### GO and KEGG enrichment analysis

**Figure 4:**
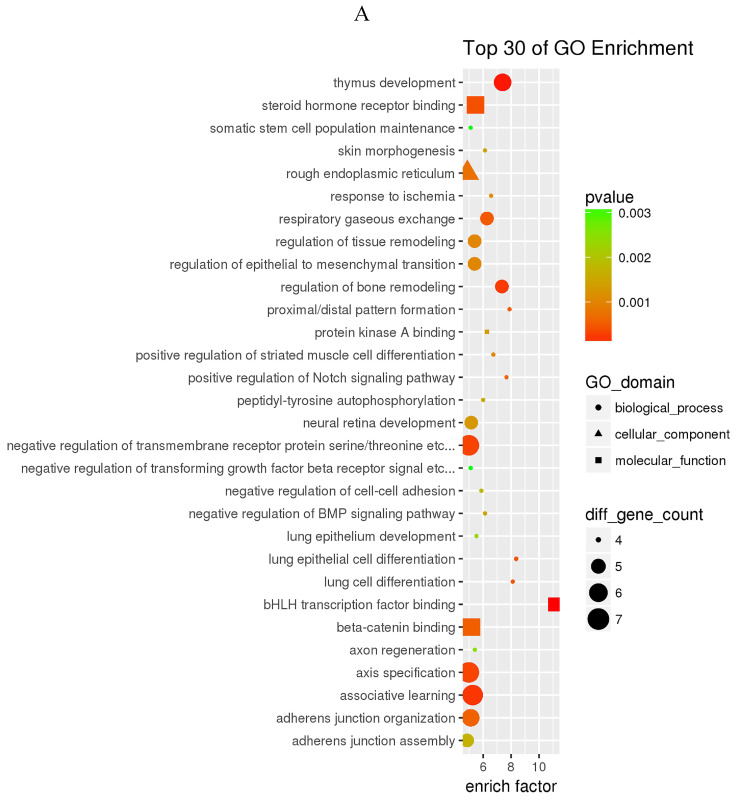

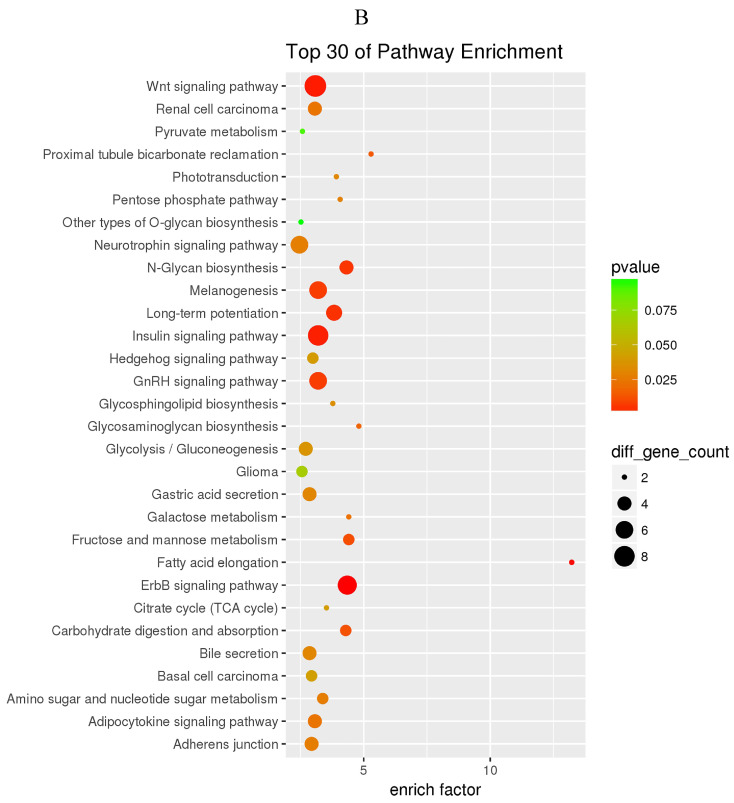

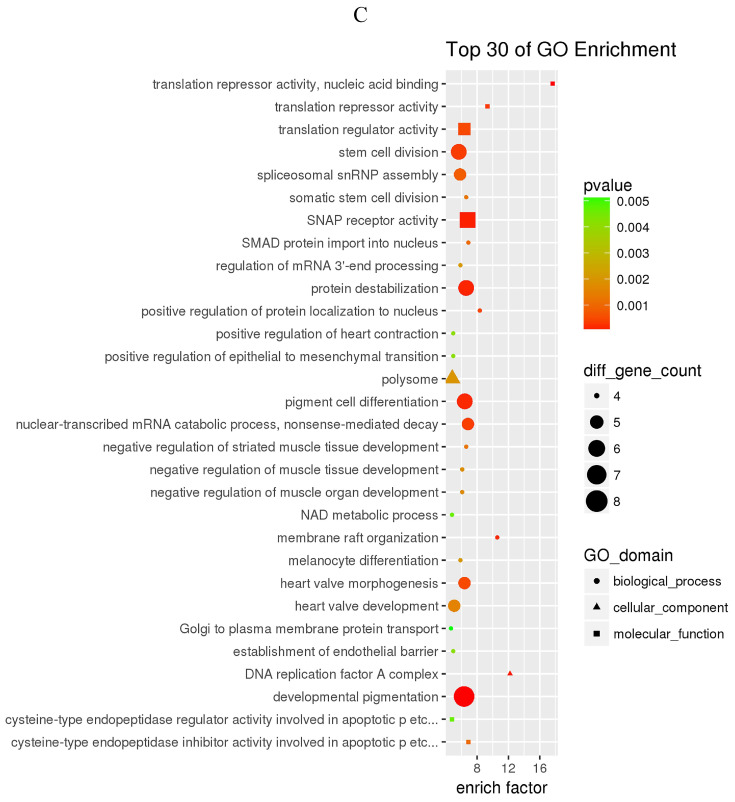

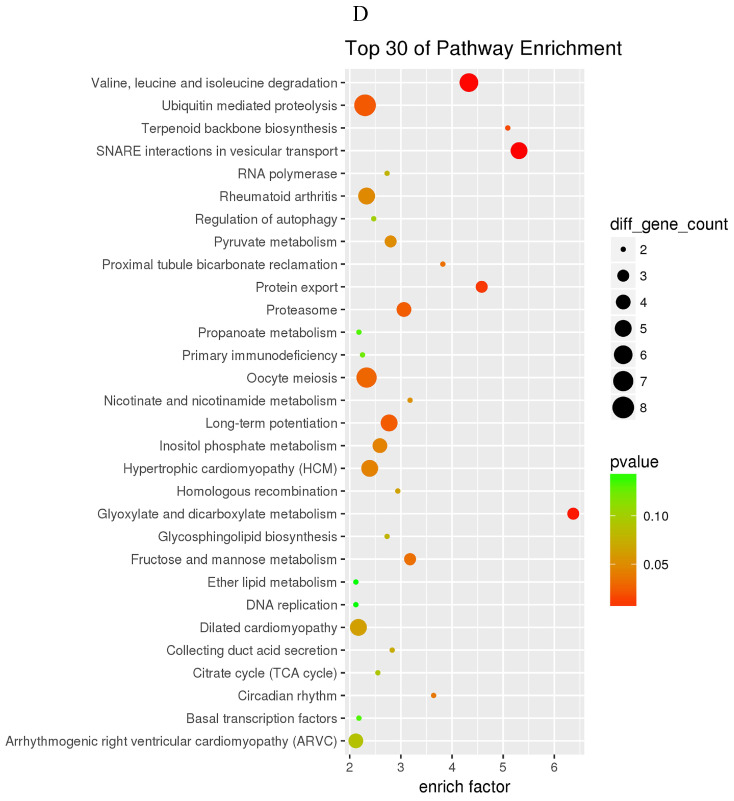
GO and KEGG enrichment analyses of the predicted target genes of miR-34a-5p and miR-452-5p. In the GO enrichment analysis figures (A and C), the circle indicated biological process, triangle indicated cellular component, and square indicated molecular function. The size of the graph indicated the number of different genes in the related item, the color indicated the significance of the difference, and the more to right of their position, the higher the level of enrichment. In the KEGG enrichment analysis figures (B and D), the size of circles indicated the number of different genes in the related pathway, the color indicated the significance of the difference, and the more to right of their position, the higher the level of enrichment.

The prediction of target genes of miR-34a-5p and miR-452-5p was based on the TargetScan and miRNA Target Prediction Database (miRDB). The results showed that the miR-34a-5p with significantly high expression in the pancreas of ZDF rats had 332 target genes, while the miR-452-5p with significantly low expression had 341 target genes. Furthermore, GO analysis of miR-34a-5p target genes revealed that their primary function was to interact with steroid hormone receptors and β-catenin (Figure 4A), while the target genes of miR-34a-5p may functionally related to the synaptosomal-associated protein (SNAP) receptor activity (Figure 4C). Moreover, the KEGG analysis showed the involvement of miR-34a-5p in multiple crucial signaling pathways, including Wnt signaling, gonadotropin-releasing hormone (GnRH) signaling, insulin signaling, and ErbB signaling pathways (Figure 4B). On the other hand, miR-452-5p was found to be involved in certain amino acid (valine, leucine and isoleucine) degradation pathways as well as the ubiquitin-proteasome pathway (UPP) for proteolysis (Figure 4D).

### The target genes of miR-34a-5p and miR-452-5p

According to the KEGG analysis results, a total of 4 signaling pathways were mainly related to miR-34a-5p expression, and they corresponded to a total of 21 target genes. Meanwhile, there were 2 signaling pathways primarily related to miR-452-5p expression, and they corresponded to 14 target genes (Table 2).

### The expression of miR-34a-5p and miR-452-5p in control vs. diabetic rat model

**Figure 5:**
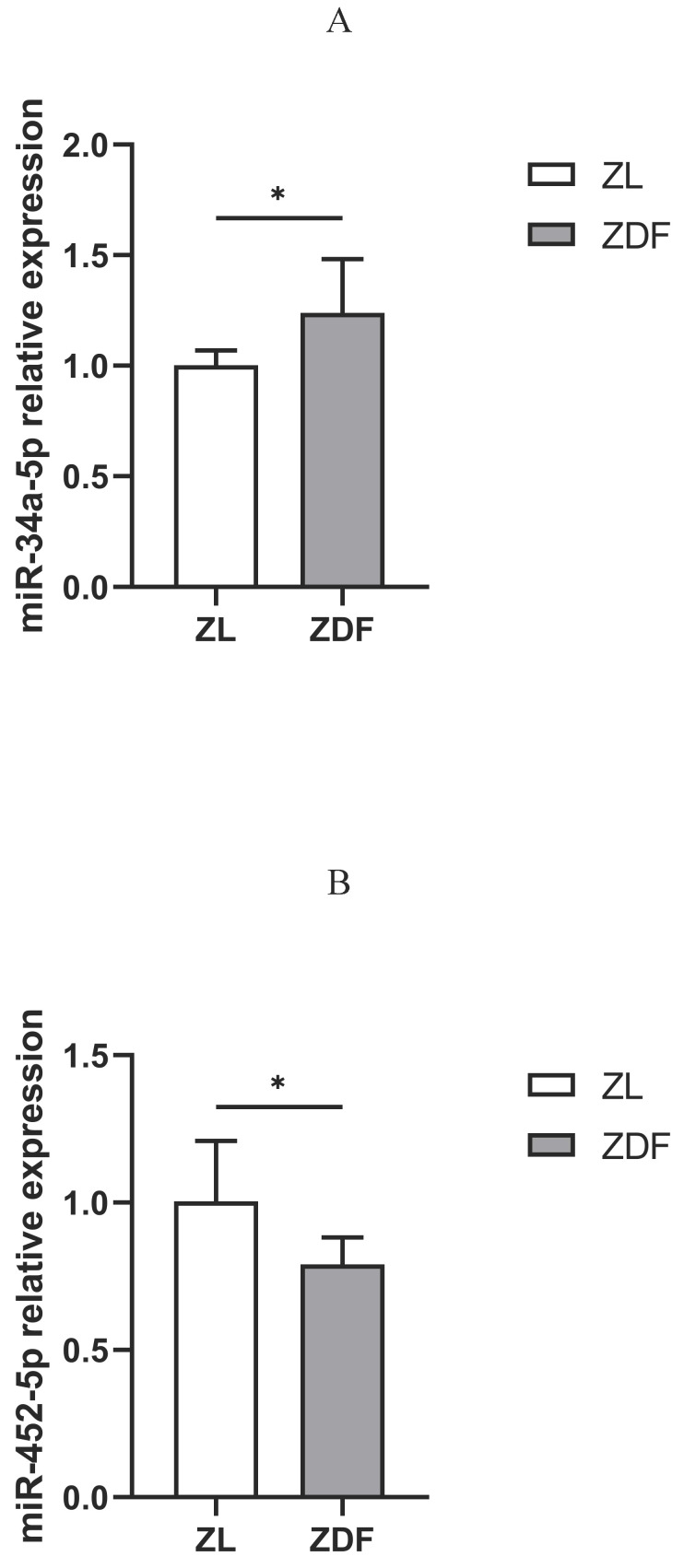
qRT-PCR analysis of miR-34a-5p and miR-452-5p expressions. Compared to ZL rats in the control group, the expression of miR-34a-5p in the pancreas of diabetic ZDF rats was up-regulated (A), while miR-452-5p expression was down-regulated (B). The data shown are representative of at least three independent experiments (n=4 for each group, 3 replicate holes for each sample) and represented as mean ±SD. The statistically significant is indicated by ^*^P<0.05.

The qRT-PCR assay revealed that the expression of miR-34a-5p was up-regulated (Figure 5A), while that of miR-452-5p was down-regulated in the pancreas of the ZDF rats compared to those in control (ZL) rats (Figure 5B).

## Discussion

Insulin is one of the critical hormones that regulate blood glucose homeostasis. The major physiological effects of insulin secretion include inhibition of hepatic gluconeogenesis and glycogenolysis, stimulation of skeletal glucose uptake, and suppression of adipose lipolysis^10^. These functions make pancreatic β-cells irreplaceable in the process of glucose metabolism. Once there is dysregulation of these physiological functions, such as delayed or decreased insulin secretion, hyperglycemia occurs.

Pancreatic endocrine β-cells function is affected by both genetics and/or environmental factors, including race, family history, age, disease course, and body mass index (BMI), etc.^11^. Patients with poor residual β-cell function are often required to administer exogenous insulin in the early stage of clinical treatment^12^. However, exogenous insulin treatment is only a mitigation strategy, since the protective effects disappear when there is no external insulin input. Also, other therapeutic medications, such as metformin^13^, dipeptidyl peptidase-IV (DPP-4) inhibitors^14^, glucagon-like peptide-1 (GLP-1) receptor agonists^15^ and sodium glucose cotransporter-2 (SGLT2) inhibitors^16^, have the protective effects on β-cells function in the short term, including improving insulin sensitivity, inducing β-cells' and inhibit α-cells' proliferation, but their long-term efficacy remains unclear. Therefore, the research on the mechanism of pancreatic endocrine β-cells' dysfunction is important.

The primary reason for the β-cell deficiency in the diabetic condition could be apoptosis and dedifferentiation^17^. However, the underlying molecular mechanisms of these deregulations have not been fully explored. MiRNAs are ubiquitously present in the eukaryotic cells, where they exercise a wide range of physiological functions. In pancreatic β-cells, miRNA maintains the balance among cell proliferation, differentiation and apoptosis^18^. When some miRNAs are overexpressed, they can induce dedifferentiation by inhibiting the expression of genes that maintain the mature phenotype of β-cells^19^, or induce β-cells apoptosis by up-regulating the expression of pro-apoptotic genes^20^, which eventually leads to diabetes.

In previous studies on miRNA profiling in rodents, most of the miRNA expression profiles were generated from peripheral blood^21^, liver and other tissues^22^. In contrast, there are only a few miRNA expression profiles generated from pancreatic tissue samples. This may be because the pancreas secretes a large number of digestive enzymes, and those enzymes exert a stronger chemical degradation effect on the pancreatic tissue, making it difficult to extract a complete and intact miRNA expression profiles from these kinds of specimens.

ZDF rat model is a spontaneous well-established diabetic model and an ideal model for observing the natural progression of T2D^23^. ZDF rats have leptin receptor defects, which makes them exhibit the characteristics of metabolic syndrome from the early stages of development, such as obesity, hyperglycemia, and hyperinsulinemia, and eventually β-cell dysfunction^24^, ^25^. In this study, we fed ZDF rats with HFD for 4 weeks to detect the pancreatic differential miRNA expression-related genes in ZDF (fa/fa) rats and ZL (fa/+) rats by microarray assays. Finally, among 24 candidate miRNAs, we found two miRNAs, miR-34a-5p and miR-452-5p, which showed the most significant regulatory effects on their target genes. These two miRNAs have previously been reportedly found in the lung^26^-^28^, liver^28^, ^29^, kidney^30^, ^31^, and a certain type of cancer cells^32^, ^33^. In recent studies, miR-34a-5p has been found to mediate the lipotoxic effect of pancreatic β-cells in hyperlipidemia mice^34^, while miR-452-5p has been reported to be involved in the tumorigenesis especially reproductive endocrine tumors such as prostate cancer^35^ and breast cancer^36^. However, miR-34a-5p and miR-452-5p expressions have never been reported in the pancreas of diabetic individuals or animal models, and there is only a handful of research investigating their relationship with blood glucose metabolism. Therefore, we examined their target genes by using GO and KEGG enrichment analyses, and further verified their expressions by qRT-PCR assay.

In the KEGG enrichment analysis of miR-34a-5p target genes, we found that the Wnt and the insulin signaling pathways-related genes were the most enriched. The GO enrichment analysis also showed that the most important molecular function of miR-34a-5p was binding with β-catenin. The above results further confirmed that miR-34a-5p was closely related to the Wnt/β-catenin signaling axis, one of the most critical links in the classic Wnt signaling pathway.

Wnt signaling pathway can affect β-cells proliferation^37^. Wnt protein is a highly conserved secreted protein. Its ligands bind and activate Frizzleds (Fz), a transmembrane receptor protein, thereby stabilizing β-catenin combined with transcription factor 7-like 2 gene (TCF7L2) and causing its nuclear translocation^38^. Dessimoz J et al.^39^ has reported that selective inactivation of β-catenin results in β-cells' hypoplasia. Struan F A Grant et al.^40^ reported that TCF7L2 plays an important physiological role both in pancreas and insulin sensitive organs, regulating the intestinal secretion of GLP-1 to induce β-cell proliferation^41^.

Based on the above results, we speculate that miR-34a-5p may serve as an antagonist to β-catenin expression, blocking the conduction of Wnt signaling cascades in the pancreas, and inhibiting the expression of a variety of cell cycle regulators and functional transcription factors in β-cells. miR-34a-5p affects the development and functional maturity of β-cells, which in turn decreases the body's tolerance to glucose level and the insulin secretion. Meanwhile, the reduced level of β-catenin down-regulates the expression of TCF7L2, affecting the expression of the proglucagon gene in the ileum, thereby reducing the secretion of GLP-1, leading to T2D onset. At the same time, we found that Wnt1 and TCF7L1, the direct target genes of miR-34a-5p, were also involved in the conduction of the Wnt signaling pathway (Table 2). TCF7L1 is a Wnt inhibitor and stem cell regulator that affects the development of embryonic stem cells^42^, suggesting that miR-34a-5p may affect the development of fetal stem cells of pancreatic lineage.

Another major pathway that miR-34a-5p is involved in is insulin signaling pathway which has the most direct regulatory effect on blood glucose homeostasis. Insulin signaling pathway mainly consists of the PI3K/Akt pathway and the ERK/MAPK pathway. The latter is a mitotic regulation pathway that causes cells proliferation and differentiation by activating specific transcription factors^43^. We found that the miR-34a-5p target gene related to the insulin signaling pathway was MAPK10. This suggests that miR-34a-5p may directly inhibit the expression of MAPK10 and reduce the activation of downstream transcription factors, thus affecting the proliferation and differentiation of β-cells.

miR-452-5p is the most down-regulated microRNA in the pancreas of the diabetic ZDF rats. The results of KEGG showed that its target genes were mostly enriched in valine, leucine, and isoleucine degradation pathway. Valine, leucine, and isoleucine all have functional R group branches, therefore, they are referred to as branched chain amino acids (BCAA)^44^. They are important nutrients and metabolic regulators that are beneficial to health. However, many studies have found that BCAAs play an important role in the pathogenesis of metabolic diseases in recent years^45^, ^46^.

Since BCAAs are essential amino acids that can only be supplemented by food, their levels in the body can only be controlled through catabolism^47^. The most important key rate-limiting enzyme in this process is branched-chain α-keto acid dehydrogenase (BCKDH)^48^, if the activity of BCKDH reduced, the rate of the entire reaction system will decreases, leading to the suppression of BCAA catabolism, which will then lead to the accumulation of excessive BCAAs in the body that cannot be fully eliminated, thus building up the concentration of toxic metabolites. This serious result will further leads to the mitochondrial dysfunction in β-cells, and ultimately leading to T2D pathology^49^. The KEGG results showed that BCKDHB was a target gene of miR-452-5p, and it has been indicated that miR-452-5p directly regulates the expression of BCKDHB gene, one of the major catalytic subunits of BCKDH. It has been further verified that the activities of BCKDH and its catalytic subunits all decrease under diabetic conditions^50^. In view of the low expression of miR-452-5p in the pancreas of ZDF rats, we are assuming that there might be a positive regulation between miR-452-5p and BCKDHB. The low expression of miR-452-5p might have inhibited the expression of the BCKDHB gene, leading to the accumulation of BCAAs in the pancreas and ultimately damaging β-cells.

In summary, there are still many miRNAs related to pancreatic endocrine β-cell dysfunction that have not yet been determined. Their functions and action mechanisms are being discovered with the development of scientific research. Therefore, we believed that our research of miR-34a-5p and miR-452-5p would be valuable in the light of understanding the miRNA-mediated regulation of T2D onset and progression.

However, there is a limitation associated with our study. We only conducted a preliminary study on these two miRNAs in vivo, and have not performed their functional verification like inhibition or overexpression. Hence, it is worth further investigating the functional implication of these miRNA in relation to diabetes in the future.

## Conclusion

We identified that miR-34a-5p and miR-452-5p were significantly differentally expressed in the pancreas of ZDF rats. We speculated that they might be the novel miRNAs involved in the etiopathogenesis of pancreatic endocrine β-cells dysfunction, and might have the potential to be new predictive biomarkers related to T2D development and progression, as well as the therapeutic targets for T2D treatment.

## Supplementary Material

Supplementary tables.

## Figures and Tables

**Table 1:** List of significant differentially expressed miRNAs in the pancreas.

MicroRNA	P-value	Fold Change	Nucleotide sequence	Genomic location	Mirbase accession_No
Up-regulated microRNAs					
rno-miR-34a-5p	0.002903	2.188879	ACAACCAGCTAAGACACTGC	chr5	MIMAT0000815
rno-miR-743b-5p	0.007534	1.032672	ATGATGGACACCAGTCT	chrX	MIMAT0017288
rno-miR-292-3p	0.012191	1.036416	ACACTCAAAACCTGGCGGC	chr1	MIMAT0000897
rno-miR-1949	0.021023	1.726167	AACTATGCTGACATCCTG	chr18	MIMAT0017852
rno-miR-3068-3p	0.023419	1.102928	TGTTGGAGTACTGCAATT	chr6	MIMAT0024846
rno-miR-122-3p	0.023779	1.034741	TTAGTGTGATGATGGCG	chr18	MIMAT0017116
rno-miR-92a-3p	0.030293	1.471097	CAGGCCGGGACAAGTGC	chrX	MIMAT0000816
rno-miR-3559-3p	0.035091	1.050094	CACGACAGACTCAGTA	chrX	MIMAT0017828
rno-miR-376a-5p	0.043960	1.079268	CTCATAGAAGGAGAATCTACC	chr6	MIMAT0003197
rno-miR-384-3p	0.045926	1.023334	ATTGTGAACAATTTCTAGGAAT	chrX	MIMAT0005310
Down-regulated microRNAs					
rno-miR-452-5p	0.000039	0.017976	CTCAGTTTCCTCTGCA	NA	MIMAT0035748
rno-miR-345-5p	0.049379	0.483844	GCACTGGACTAGGGGT	chr6	MIMAT0000594
rno-miR-16-5p	0.017979	0.586664	CGCCAATATTTACGTGCTG	chr2	MIMAT0000785
rno-miR-181d-5p	0.009535	0.631897	ACCCACCGACAACAATG	chr19	MIMAT0005299
rno-miR-499-5p	0.008271	0.904235	AAACATCACTGCAAGTCTT	chr3	MIMAT0003381
rno-miR-378b	0.029414	0.910314	CCTTCTGACTCCAA	chr5	MIMAT0024855
rno-miR-325-5p	0.019239	0.929482	ACACTTACTGAGCACCTA	chrX	MIMAT0000557
rno-miR-127-5p	0.012196	0.944294	AATCAGAGCCCTCTGAG	chr6	MIMAT0017117
rno-miR-880-3p	0.019889	0.947277	TCTACTCAGAATGAATGGAGT	chrX	MIMAT0005288
rno-miR-323-3p	0.043819	0.972565	AGAGGTCGACCGTGTAATGT	chr6	MIMAT0000550
rno-miR-363-5p	0.028662	0.973161	AAATTGCATCGTGATCCAC	chrX	MIMAT0003209
rno-miR-412-3p	0.026106	0.974151	ACGGCTAGTGGACCAG	chr6	MIMAT0003124
rno-miR-6334	0.005202	0.980032	GCCGGCAGCTGG	chr3	MIMAT0025075
rno-miR-301b-5p	0.042853	0.985857	AGTAGTGCAACCTAGTCA	chr11	MIMAT0017300

There are 6 parameters in the table, including P-value, fold change, nucleotide sequence, chromosome location and miRBase number. MiRNA expression difference was considered significant when P<0.05, and fold change≤0.5 or fold change≥2.

**Table 2:** The pathway and target genes of miR-34a-5p and miR-452-5p

PathwayID	Pathway Description	P-value	Target Genes
miR-34a-5p			
rno04310	Wnt signaling pathway	0.002230	DAAM2 MAPK10 PLCB2 PPP2CA TCF7L1 CAMK2B WNT7A WNT1 CAMK2A
rno04910	Insulin signaling pathway	0.002749	MAPK10 PPP1CC CRKL PRKAG1 LIPE HK1 PCK1 MKNK1
rno04912	GnRH signaling pathway	0.007240	MAP2K3 FSHB MAPK10 PLCB2 CAMK2B CAMK2A
rno04012	ErbB signaling pathway	0.000649	MAPK10 CRKL PAK6 CAMK2B TGFA ABL1 CAMK2A
miR-452-5p			
rno00280	Valine, leucine and isoleucine degradation	0.001316	BCKDHB ALDH6A1 ACAT1 ACADSB DBT MCCC2
rno04120	Ubiquitin mediated proteolysis	0.021330	UBA3 PRPF19 SIAH1 ANAPC11 UBE2G1 UBA6 UBE2D1 XIAP

## Abbreviations

T2D: Type 2 diabetes
miRNA: microRNA
ZDF: Zucker diabetic fatty
ZL: Zucker lean
HFD: high-fat diet
NFD: normal-fat diet
qRT-PCR: quantitative real-time polymerase chain reaction
GO: Gene Ontology
KEGG: Kyoto Encyclopedia Genes and Genomes
BCAA: branched chain amino acids
BCKDHB: branched-chain α-keto acid dehydrogenase-β
RISC: RNA-induced silencing complex
FFA: free fatty acids
TCF7L2: transcription factor 7-like 2 gene
TCF7L1: transcription factor 7-like 1 gene
DPP-4: dipeptidyl peptidase IV
GLP-1: glucagon-like peptide-1
SGLT2: Sodium glucose cotransporter 2
MAPK: mitogen-activated protein kinase
PI3K: phosphatidylinositol 3-kinase
Akt: protein kinase B
ERK: Extracellular signal-regulated kinase
