# Supplementary material for: MiR-34a-5p and miR-452-5p: The Novel Regulators of Pancreatic Endocrine Dysfunction in Diabetic Zucker Rats?
Source: Int J Med Sci. 2021 Jul 11;18(14):3171–81. doi: 10.7150/ijms.62843 (PMC8364455; doi:10.7150/ijms.62843)
Supplement: Supplementary file 1 — Supplementary tables. [file ijmsv18p3171s1.pdf]

Table S1. Effect of HFD-feed on body weights in ZDF rats.

| Group | n | Week 0 (g) | Week 1 (g) | Week 2 (g) | Week 3 (g) | Week 4 (g) |
|---|---|---|---|---|---|---|
| Control | 5 | 266.67±5.74 | 280.30±6.46 | 295.10±6.15 | 320.41±6.83 | 331.20±9.11 |
| Model | 5 | 354.03±11.42*** | 383.12±32.31*** | 398.74±35.01*** | 428.75±54.75*** | 435.10±63.48*** |

**Table S1.** Effect of HFD-feed on body weights in ZDF rats. Data are represented as mean ± SD. Statistically significant between control group and experimental group were indicated by ***P<0.001.

Table S2. Effect of HFD-feed on fasting glucose in ZDF rats.

| Group | n | Week 0 (mmol/L) | Week 1 (mmol/L) | Week 2 (mmol/L) | Week 3 (mmol/L) | Week 4 (mmol/L) |
|---|---|---|---|---|---|---|
| Control | 5 | 3.32±0.15 | 3.30±0.32 | 3.58±0.25 | 3.30±0.09 | 3.62±0.16 |
| Model | 5 | 14.82±2.50*** | 17.80±2.69*** | 19.20±5.47*** | 22.56±3.77*** | 25.66±2.85*** |

**Table S2.** Effect of HFD-feed on fasting glucose in ZDF rats. Data are represented as mean ± SD. Statistically significant between control group and experimental group were indicated by ***P<0.001.

Table S3. Primer sequences of qRT-PCR assay

| Gene | | Primer sequences |
|---|---|---|
| miR-34a-5p | Stem loop primer | 5'-GTCGTATCCAGTGCGTGTCGTGGAGTCGGCAATTGCACTGGATACGACGCAGTG-3' |
| | Forward primer | 5'-CGTCCACAACCAGCTAAGA-3' |
| | Reverse primer | 5'-GCAGGGTCCGAGGTATTC-3' |
| miR-452-5p | Stem loop primer | 5'-GTCGTATCCAGTGCGTGTCGTGGAGTCGGCAATTGCACTGGATACGACTGCAGA-3' |
| | Forward primer | 5'-CGGCCTCAGTTTCCTCTGCA-3' |
| | Reverse primer | 5'-GCAGGGTCCGAGGTATTC-3' |
| U6 | Forward primer | 5'-CTCGCTTCGGCAGCACATATACT-3' |
| | Reverse primer | 5'-ACGCTTCACGAATTTGCGTGTC-3' |

**Table S3.** Primer sequences of qRT-PCR assay. MiR-34a-5p and miR-452-5p both use

special stem-loop primers at reverse transcription, and U6 is the internal reference gene.
